# Correction: Extracellular histones are clinically associated with primary graft dysfunction in human liver transplantation

**DOI:** 10.1039/c9ra90059d

**Published:** 2019-08-22

**Authors:** Xiuhui Li, Chunyan Gou, Yanhua Pang, Yakun Wang, Yan Liu, Tao Wen

**Affiliations:** Department of Liver Diseases, Beijing Youan Hospital, Capital Medical University Beijing 100069 P. R. China; Department of Gastroenterology, Beijing Chaoyang Hospital, Capital Medical University Beijing 100020 P. R. China; Medical Research Center, Beijing Chao-Yang Hospital, Capital Medical University Beijing 100020 P. R. China wentao5281@163.com

## Abstract

Correction for ‘Extracellular histones are clinically associated with primary graft dysfunction in human liver transplantation’ by Xiuhui Li *et al.*, *RSC Adv.*, 2019, **9**, 10264–10271.

The e-mail contact address for the corresponding author Tao Wen, was omitted in the published article and is shown here.

The Royal Society of Chemistry apologises for these errors and any consequent inconvenience to authors and readers.

## Supplementary Material

